# Impact of a transition education program on health-related quality of life in pediatric patients with congenital heart disease: study design for a randomised controlled trial

**DOI:** 10.1186/s12955-021-01668-1

**Published:** 2021-01-19

**Authors:** Oscar Werner, Charlene Bredy, Kathleen Lavastre, Sophie Guillaumont, Gregoire De La Villeon, Marie Vincenti, Cristelle Gerl, Yves Dulac, Nathalie Souletie, Philippe Acar, Laurence Pages, Marie-Christine Picot, Gerard Bourrel, Agnes Oude Engberink, Elodie Million, Hamouda Abassi, Pascal Amedro

**Affiliations:** 1 Pediatric and Congenital Cardiology Department, M3C Regional Reference CHD Centre, University Hospital, 371 Avenue du Doyen Giraud, 34295 Montpellier, France; 2 Pediatric Cardiology and Rehabilitation Unit, St-Pierre Institute, Palavas-Les-Flots, France; 3 Pediatric and Congenital Cardiology Department, M3C Regional Reference CHD Centre, Toulouse University Hospital, Toulouse, France; 4 Epidemiology and Clinical Research Department, University Hospital, Montpellier, France; 5 Clinical Investigation Centre, INSERM–CIC 1411, University of Montpellier, Montpellier, France; 6 Department of General Medicine, University of Montpellier, Montpellier, France; 7 PhyMedExp, INSERM, CNRS, University of Montpellier, Montpellier, France

**Keywords:** Transition, Therapeutic education, Congenital heart disease, Quality of life, Adolescent

## Abstract

**Background:**

Recent advances in the field of congenital heart disease (CHD) led to an improved prognosis of the patients and in consequence the growth of a new population: the grown up with congenital heart disease. Until recently, more than 50% of these patients were lost to follow up because of the lack of specialized structures. The critical moment is the transition between paediatric and adult unit. Therapeutic education is crucial to solve this issue by helping patients to become independent and responsible. The TRANSITION-CHD randomized trial aims to assess the impact of a transition education program on health-related quality of life (HRQoL) of adolescents and young adults with CHD.

**Methods:**

Multicentre, randomised, controlled, parallel arm study in CHD patients aged from 13 to 25 years old. Patients will be randomised into 2 groups (education program vs. no intervention). The primary outcome is the change in self-reported HRQoL between baseline and 12-month follow-up. A total of 100 patients in each group is required to observe a significant increase of the overall HRQoL score of 7 ± 13.5 points (on 100) with a power of 80% and an alpha risk of 5%. The secondary outcomes are: clinical outcomes, cardiopulmonary exercise test parameters (peak VO2, VAT, VE/VCO2 slope), level of knowledge of the disease using the Leuven knowledge questionnaire for CHD, physical and psychological status.

**Discussion:**

As the current research is opening on patient related outcomes, and as the level of proof in therapeutic education is still low, we sought to assess the efficacy of a therapeutic education program on HRQoL of CHD patients with a randomized trial.

***Trial registration*:**

This study was approved by the National Ethics Committee (South-Mediterranean IV 2016-A01681-50) and was registered on Clinicaltrials.gov (NCT03005626).

## Background

Congenital heart diseases (CHD) are the first cause of congenital abnormality at birth. As a result of significant improvements in surgical techniques and intensive care, more adults than children are currently living with a CHD [1–4]. However, young patients with a CHD are prone to interrupt their follow-up soon after transfer from paediatric to adult cardiology and are therefore at risk to reintegrate the healthcare system when facing a complication [5, 6]. Indeed, more than 30% of adults with a CHD are lost to follow-up despite a significant risk of arrhythmia, heart failure, pulmonary hypertension and acquired cardiovascular diseases [7, 8]. Even patients with lower-complexity CHD are concerned with a higher burden of adverse cardiovascular events relative to the general population [7]. Some lost to follow-up adult patients with a CHD reintegrate the healthcare system for administrative issues: they may be referred to an expert CHD centre by an occupational medicine physician, or when taking out a bank loan by an insurance service. Lost to follow-up female CHD patients are frequently referred by obstetricians in early pregnancy.

This public health issue has been integrated in the recent European and American CHD guidelines, which emphasized the interest of structured transition education programs dedicated to adolescents and young adults with CHD, before transfer to adult care [9, 10]. Transition education programs intend to improve patients’ knowledge of their medical condition, and answer their questions about physical activity, sexuality, graduate studies, and professional carriers [6, 11]. Those programs aim to promote patient autonomy in order to limit loss to follow-up, and, ultimately, improve medical care.

Various transition education programs have being experimented worldwide in patients with CHD, however, their real impact on patient care has been scarcely evaluated [5, 6, 11–13]. Heterogeneity in terms of type of education, disease severity and patient demographic characteristics may result in biased interpretation on the actual program’s efficacy. Moreover, patient reported outcomes assessment such as health-related quality of life (HRQoL), and qualitative analyses, should be part of such education programs’ evaluation [14–18].

Most importantly, randomised controlled trials are necessary to increase the level of evidence in the field of CHD patient education [19, 20].

In a pilot study evaluating the implementation of our own transition program, we showed that patients with complex CHD, poor disease knowledge, risk behaviours, and lower age, were more likely to join this transition program [6]. However, these preliminary results come from a non-randomized controlled study. Therefore, the impact of this transition program needs to be determined with a higher level of evidence.

In the TRANSITION-CHD trial, we aim to assess the impact of a non-selective structured education transition program on HRQoL of adolescents and young adults with CHD, through a regional multicentre randomised trial. We also intend to perform a qualitative phenomenological study to explore how these young patients experience the TRANSITION-CHD program and identify key factors for success or failure.

## Methods

### Study design

The TRANSITION-CHD trial is an open label prospective, multicentre, randomised, controlled, parallel arm study. Patients will be followed for 12 months with an expected recruitment time period of 24 months.

**Fig. 1:**
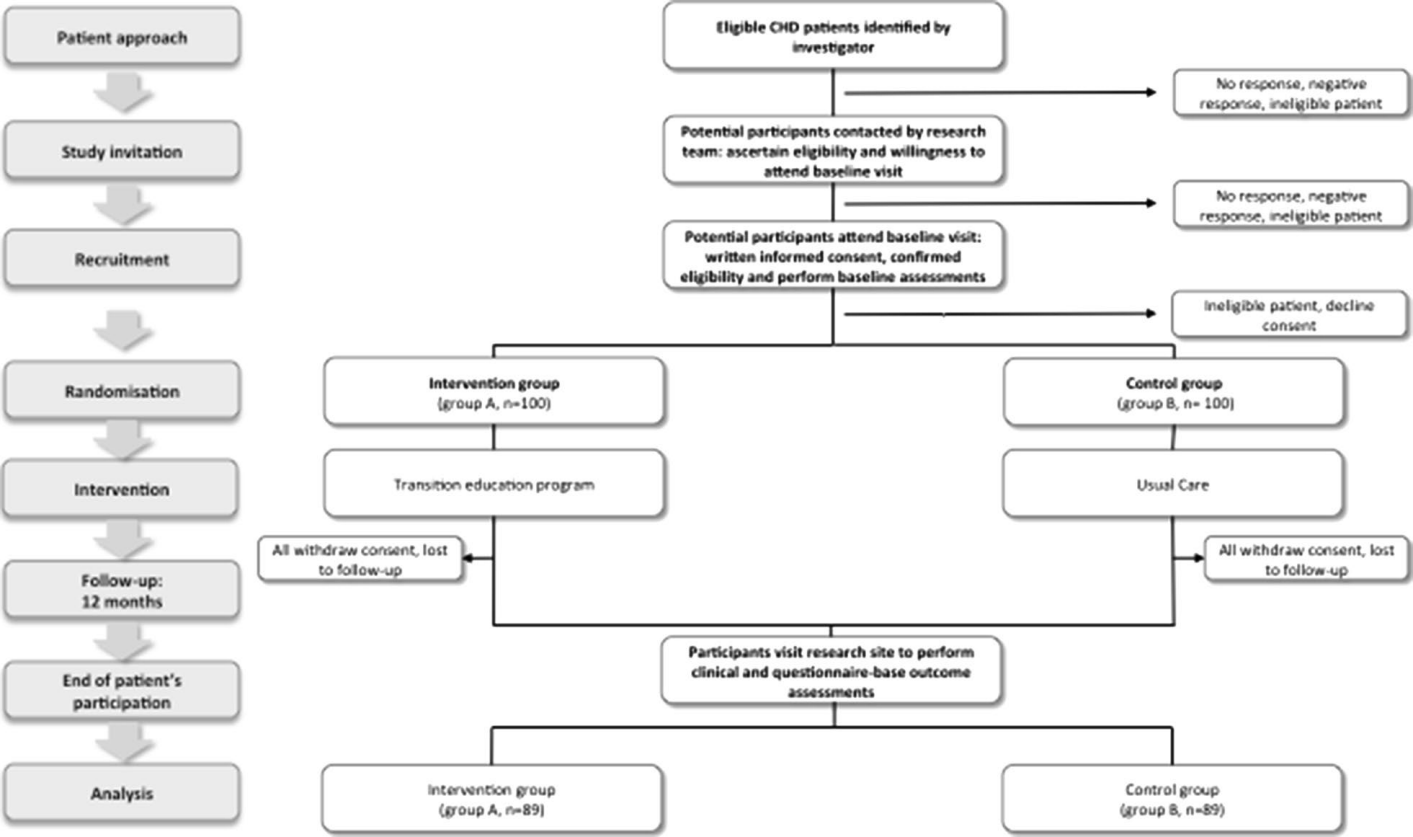
Flow chart

Once the informed consent will be given, participants will be randomly allocated in a 1:1 ratio to either intervention (e.g. transition education program) or control group arms. In the control group, patients will have a regular non-modified follow-up with no formalized transition education program during the 12-month study period. However, they will be offered to participate in the transition therapeutic education program, once the 12-month study period is over (Fig. 1).

Randomisation will be stratified by age group (13–17 years and 18–25 years), centralised, using a secure, web-based randomisation system (Ennov Clinical Software) managed by the Clinical Research Unit of Montpellier University Hospital, independently from the investigators.

### Setting

Overall, 3 CHD centres in the south of France will participate in the study (Montpellier University Hospital, Saint-Pierre Institute, Toulouse University Hospital). A total of 17 investigators will oversee patient recruitment. These investigators are cardiac surgeon (n = 1), paediatric cardiologists (n = 11), adult congenital cardiologists (n = 2) or both (n = 3). Patients will be recruited in tertiary care centres labelled by health authorities as referral centres for complex congenital heart diseases (e.g. “M3C” national health network).

### Funding

Montpellier university hospital is the sponsor of the TRANSITION-CHD trial. The study was funded by an institutional young researcher award from Montpellier University Hospital (AOI-2016), a paramedical research award from the French Department of Health (GIRCI-SOHO-APIRES-2017), and a research fellow grant from the French Society of Cardiology (Bourse Hélène de Marsan 2017-FCPC-SFC). The funding sources will have no involvement in any part of the research.

### Study population

Patients with a CHD, as defined by the international ACC-CHD classification, [21] and aged from 13 to 25 years old, will be prospectively recruited in the participating centres, during an outpatient visit. Inclusion and exclusion criteria were listed in Table 1.

**Table 1:** Trial entry

*Inclusion criteria*
Male or female aged 13–25 years old
Patients with a congenital heart disease (CHD), as defined by the international ACC-CHD classification
Written informed consent for adult patients, or legal guardians and children’s assent for minors
*Exclusion criteria*
Patients who are unable to understand the study information or unable to complete study procedures
Patients with a severe intellectual disability that does not allow the completion of the quality of life questionnaire
Patient who intends to move to another region with a follow-up in another institution during the 12 month study period
Patients participating in concurrent interventional research which may overburden the patient or confound data collection

### Intervention

As previously described, the structure of our transition program [6] involves all educational objectives described in current guidelines on transition care in patients with CHD [9, 10]. The transition education program is divided into three parts, as follows:

1. *First educational outpatient visit (1 h):* this individual interview with a health educator (e.g. a specialist nurse) aims to determine the patient’s educational objectives and needs.
2. *Group session (1 day):* dedicated to patients and their relatives, this group session with 5 to 8 patients of similar age ranges (13–17 years or 18–25 years) and involves two health educators, one paediatric cardiologist, one adult congenital cardiologist, one psychologist and one patient association delegate. The main goal is to promote patient’s self-efficacy and autonomy. The program of the day is divided into four parts:
  - *Medical aspects:* knowledge of the disease, type of repair, follow-up in adult cardiology, and complications.
  - *“Living with a CHD”:* interactive talkgroup (youth and parents separately) including various education tools and addressing several topics (sports, jobs, studies, sexuality, etc.). For the patients, the main goal is to improve their self-advocacy in deciding to act both independently from parents and medical providers, and in interdependence with them. Specific messages to promote physical activity and limit sedentary lifestyle in patients with CHD are delivered by the educational team. For the parents, the main goal is to identify factors that could promote or inhibit their child’s autonomy.
  - *Administrative workshop:* group session (patients and relatives together) addressing various following topics such as social security, insurance or bank loans.
  - *Synthesis and individual interview*: individual interview with a cardiologist and a health educator, to establish a personalized educational report. When needed, additional healthcare and educational objectives are defined, such as psychological support, knowledge reinforcement, cardiac rehabilitation, etc.
3. *Transfer preparation outpatient visit*: approximately 6 months after the group session, the patient undergo a medical visit with both a paediatric cardiologist and an adult congenital cardiologist, to provide an individual feedback from the group session and to prepare the transfer in the adult care CHD centre.

### Main outcome

The main outcome is the change between baseline (M0) and 12-month follow-up (M12) of the PedsQL self-reported HRQoL score. The PedsQL generic HRQoL questionnaire has four multidimensional scales: physical functioning (8 items), emotional functioning (5 items), social functioning (5 items), and school functioning (5 items). The three summary scores are: total scale score (23 items), physical health summary score (8 items) and psychosocial health summary score (15 items). Each item uses a 5-point Likert scale from 0 (never) to 4 (almost always). Items are reversed scored and linearly transformed to a 0–100 scale, higher scores indicating a better HRQoL. Psychometric properties showed reliability, validity and responsiveness to clinical change over time [22]. After translation and cultural adaptation, the psychometric properties of the French version of the PedsQL appeared to be acceptable [23]. Two versions of the PedsQL questionnaire (13–17 and 18–25 years old) will be used for adolescents and young adults, respectively.

### Secondary outcomes (Table 2)

The following outcomes will be measured at baseline (M0) and 12-month follow-up (M12) and their changes over time will be analysed:

- Exercise capacity variables measured by a cardio-pulmonary exercise test (CPET): peak oxygen uptake (pVO2), ventilatory anaerobic threshold (VAT), and ventilatory efficiency (VE/VCO2 slope). As detailed in our previous trials involving exercise capacity assessment, CPET procedures in all participating laboratories will be harmonized [14, 15, 24].
- The level of physical activity with the Ricci and Gagnon questionnaire, composed by 8 items (total score < 16 points: no activity; 17–32 points: moderate activity; 33–40 points: intensive activity) [25].
- The level of knowledge with the Leuven knowledge questionnaire for CHD [26].
- The clinical outcomes: NYHA functional class, healthcare usage (primary and secondary care contacts, hospitalisation), and medication.
- The level of anxiety with the self-administered State and Trait Anxiety Inventory (STAI) questionnaire for young adults and the STAI-Children questionnaire for adolescents [27].
- The level of depression with the self-administered Beck Depression Inventory (BDI) questionnaire for young adults and the Child Depression Inventory (CDI) questionnaire for adolescents [28, 29].
- The parents-reported HRQoL score with the proxy version of the PedsQL for adolescents (aged 13–17 years old) [22].

**Table 2:** Outcome measures

*Primary outcome*
Health-related quality of life score: PedsQL self-questionnaire (version 13–18 years for adolescents and version 18–25 years for young adults)
*Secondary outcomes*
Proxy version of the PedsQL for parents of adolescents (13–18 years old)
Level of anxiety (STAI self-questionnaire for young adults and the STAI-Children self-questionnaire for adolescents)
Level of depression (BDI self-questionnaire for young adults and CDI self-questionnaire for adolescents)
Level of physical activities (RICCI and GAGNON self-questionnaire)
Cardiopulmonary exercise tests parameters (peak VO2, VAT, VE/VCO2 slope)
Clinical outcomes: NYHA functional class, blood pressure, healthcare usage (primary and secondary care contacts, hospitalisation), and medication
The socio-economic status of the patient and/or the family (only at baseline)
Acceptability of the intervention to participants
Qualitative analysis (sample of patients from the intervention group)

*STAI* state-trait anxiety inventory, *BDI* beck depression inventory, *CDI* Children’s depression inventory, *VAT* ventilatory anaerobic threshold

The acceptability of the intervention (e.g. the transition program) by the participants will also be analysed at 12 months.

The phenomenological qualitative study will be performed on a sample of patients from the group 1, after they completed the education program. Semi structured face-to-face individual interviews will be conducted by a psychologist trained in qualitative clinical research (LP), using a standardised phenomenological interview guide focussing on lived experience. Purposive sampling will be used to obtain diversity of experiences across various individual and clinical characteristics (gender, age, type of CHD, drugs, type of surgery, etc.). Data saturation principle will be chosen, without predefining the number of interviews. The data collection method will comprise a phenomenological. The disclosure of the disease and its representation will be explored, as well as the influence of the CHD in everyday life in terms of relationship to the body, lifestyle, and identity. Patients’ feeling about the transition program and its influence on their future will be explored.

### Sample size

The primary outcome is the change in the self-reported HRQoL score with the PedsQL instrument. In our previous HRQoL cross-sectional studies in patients with CHD [14, 30, 31] as well as in similar studies using patient related outcomes, a difference of less than 5 points seems irrelevant and a difference of more than 10 points is ideal, but rarely obtained in clinical trials [32, 33]. Therefore, we hypothesized to observe an increase in the overall HRQoL score of 7 ± 13.5 points (over 100). With a 80% power, a bilateral alpha risk of 5%, and potentially 20% of loss to follow-up and/or missing data on the primary outcome, we need to include 100 patients in each group.

### Statistical analysis

All included subjects will be considered in the description of the population. An intention-to-treat analysis will be used, and each randomized subject will be analysed in his/her treatment arm. A per-protocol analysis, including all randomized subjects with a valid primary efficacy measurement and with no important protocol deviation (patients who have successfully completed the transition program) that could affect the evaluation of the main outcome, will also be carried out for parameters to assess the impact of the program when compliance is good (> 80% participation in sessions).

A description of the baseline characteristics of each group will be made by giving the frequencies of the different categories for the qualitative variables and the mean with standard deviation or median with interquartile range for continuous variables. In case of non-comparability of the groups, an adjustment or stratification (in case of interaction) on the potential confounding factors will be considered in a sensitivity analysis.

The change in the self-reported HRQoL total score, as well as the changes of the 2 summary and the 4 dimensions scores, will be compared between the two groups using student test or Man-Whitney test, according to the distributions. The effect size will be calculated using Cohen’s d and its 95% confidence limits.

The statistical significance will be set at 0.05 and analyses will be performed using Statistical Analysis Systems version 9 (SAS Institute, Cary, NC, USA).

#### Ethics

The study will be conducted in compliance with the Good Clinical Practices protocol and Declaration of Helsinki principles. It was approved by a drawn National Ethics Committee (South-Mediterranean IV 2016-A01681-50), and registered on Clinicaltrials.gov (NCT03005626). Informed consent will be obtained from all patients and their parents or legal guardians for minors. Children’s assent to participate in the study will also be required.

## Discussion

In the continuity of our clinical research program on HRQoL in patients with CHD [6, 14, 30–32, 34], we expect to observe an improvement of HRQoL in patients undergoing the transition education program. Patient-reported outcomes (PRO) have been increasingly used in clinical research [35], therefore, using HRQoL as a primary outcome in the TRANSITION-CHD trial seems particularly adapted to such a therapeutic education program. Indeed, as defined by the World Health Organization, “therapeutic education should try to give and maintain the necessary competences to handle chronic disease with the objective of improving patient HRQoL”.

Currently, the level of evidence of research in therapeutic education needs to be improved, despite all methodological constraints and biases inherent to qualitative research: various qualitative outcomes, heterogeneous educational content, ethical issues related to randomisation, program based on voluntary patient participation, etc. [36]. The TRANSITION-CHD trial will use several quantitative instruments assessing various aspects of the patient well-being: self and proxy-reported HRQoL, anxiety, depression, level of autonomy and disease knowledge. In order to limit the level of content heterogeneity, this education program is based on a single and joint structure between all tertiary care CHD centres from the same region [6]. Therefore, ethical issues have been considered in the study design, and patients randomised in the control group will be offered to participate in the transition program, without any additional cost, after the 12-month study period. Since the creation of the program in 2015, all paediatric and adult congenital cardiologists from the regional M3C network have been particularly motivated to enrol their patients and participate themselves in the education sessions. Active support from all patient organisations has played an important role in promoting the program, as shown in several videos dedicated to young patients with CHD (https://www.youtube.com/watch?v=OJ67oIm0Wv8).

Promotion of physical activity is a major educational objective of the transition program. Indeed, exercise capacity in the CHD population may be impaired and many adolescents with a sedentary lifestyle are trapped in the vicious circle of physical deconditioning [15, 24, 31, 34]. Similar therapeutic education messages focusing of sports participation have been integrated into cardiac rehabilitation programs [15].

This transition program also focuses on cardiovascular and non-cardiovascular risk factors to avoid or limit in this young population. As we recently showed, many adolescents and young adults with a CHD are concerned with risk behaviours (tobacco, alcohol, drugs) with a similar magnitude as in the general population, but they are most probably at a much higher risk or cardiovascular morbidity during adulthood [6, 7, 37].

The CHD-TRANSITION trial is actively supported by health authorities and the results will hopefully help promoting the implementation of similar programs in other regions, as well as in other chronic diseases affecting teenagers and young adults. We expect to confirm that the supervision of the program by a specialist nurse as “transition care manager” is probably an interesting operating model to follow [38–40].

## Data Availability

All data generated during this study will be included in the results published article.

## Abbreviations

CHD: Congenital heart disease
CPET: Cardiopulmonary exercise test
pV02: Peak oxygen uptake
HRQoL: Health-Related Quality of Life
